# Extensive genetic differentiation detected within a model marsupial, the tammar wallaby (*Notamacropus eugenii*)

**DOI:** 10.1371/journal.pone.0172777

**Published:** 2017-03-03

**Authors:** Mark D. B. Eldridge, Emily J. Miller, Linda E. Neaves, Kyall R. Zenger, Catherine A. Herbert

**Affiliations:** 1 Australian Museum Research Institute, Sydney, New South Wales, Australia; 2 Department of Biological Sciences, Macquarie University, New South Wales, Australia; 3 School of Biological, Earth and Environmental Sciences, University of New South Wales, Kensington, New South Wales, Australia; 4 Royal Botanic Garden Edinburgh, Edinburgh, United Kingdom; 5 College of Science and Engineering and Centre of Sustainable Tropical Fisheries and Aquaculture, James Cook University, Townsville, Queensland, Australia; University of British Columbia Okanagan, CANADA

## Abstract

The tammar wallaby (*Notamacropus eugenii*) is one of the most intensively studied of all macropodids and was the first Australasian marsupial to have its genome sequenced. However, comparatively little is known about genetic diversity and differentiation amongst the morphologically distinct allopatric populations of tammar wallabies found in Western (WA) and South Australia (SA). Here we compare autosomal and Y-linked microsatellite genotypes, as well as sequence data (~600 bp) from the mitochondrial DNA (mtDNA) control region (*CR*) in tammar wallabies from across its distribution. Levels of diversity at autosomal microsatellite loci were typically high in the WA mainland and Kangaroo Island (SA) populations (*A* = 8.9–10.6; *He* = 0.77–0.78) but significantly reduced in other endemic island populations (*A* = 3.8–4.1; *He* = 0.41–0.48). Autosomal and Y-linked microsatellite loci revealed a pattern of significant differentiation amongst populations, especially between SA and WA. The Kangaroo Island and introduced New Zealand population showed limited differentiation. Multiple divergent mtDNA *CR* haplotypes were identified within both SA and WA populations. The *CR* haplotypes of tammar wallabies from SA and WA show reciprocal monophyly and are highly divergent (14.5%), with levels of sequence divergence more typical of different species. Within WA tammar wallabies, island populations each have unique clusters of highly related *CR* haplotypes and each is most closely related to different WA mainland haplotypes. Y-linked microsatellite haplotypes show a similar pattern of divergence although levels of diversity are lower. In light of these differences, we suggest that two subspecies of tammar wallaby be recognized; *Notamacropus eugenii eugenii* in SA and *N*. *eugenii derbianus* in WA. The extensive neutral genetic diversity and inter-population differentiation identified within tammar wallabies should further increase the species value and usefulness as a model organism.

## Introduction

The study of Australia’s unique marsupial fauna, continues to offer valuable insights into multiple fields including evolutionary genetics [1] and conservation biology [2]. The tammar wallaby (*Notamacropus eugenii*) (see [3,4] regarding the change of genus from *Macropus*) is one of the most intensively studied marsupials, and has become a significant model species for reproductive, developmental, physiological, immunological, ecological and genetic research (see [1,5,6–12]. The tammar wallaby was therefore the obvious candidate to be the first Australasian marsupial, and only the second marsupial species, to have its genome sequenced [1,13]. Despite this new found wealth of genomic knowledge, comparatively little is currently known about the distribution and abundance of genetic diversity within and amongst allopatric tammar wallaby populations.

**Fig 1:**
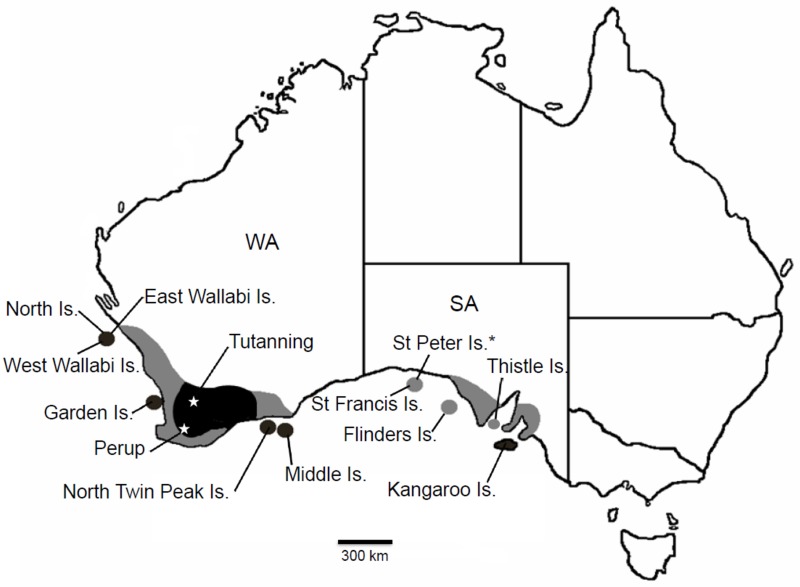
Former and current distribution of the tammar wallaby (*Notamacropus eugenii*) in Southern Australia (after [14]). Collection localities and sites mentioned in the text are indicated. Dark shading represents extant distribution; light shading represents areas where now extinct. SA = South Australia; WA = Western Australia. * = type locality. East Wallabi, West Wallabi and North Islands are part of the Houtman Abrolhos Archipelago; Middle and North Twin Peak Islands are in the Recherché Archipelago; St Francis and St Peter Islands are in the Nuyts Archipelago; Flinders Island is in the Investigator Group.

The tammar wallaby is a medium-sized macropodid (4–10 kg) with a naturally disjunct distribution across semi-arid southern Australia (Fig 1) [14]. At the time of European settlement, the tammar wallaby was distributed on the South Australian (SA) mainland and in south-western Western Australia (WA), as well as on five SA and five WA continental islands (Fig 1) [15]. In SA they have subsequently become extinct on the mainland and on four islands (Flinders, Thistle, St Francis, St Peter Islands, the latter being the type locality), and now only remain on Kangaroo Island (Fig 1), where they are abundant [15,16]. In WA, tammar wallabies have also declined on the mainland but remnant populations survive at several sites with dense native vegetation and are now recovering under ongoing fox control [17]. Tammar wallabies are still extant on all five WA islands; East and West Wallabi Islands in the Houtman Abrolhos Archipelago, Middle and North Twin Peak Islands in the Recherché Archipelago and Garden Island (Fig 1, Table 1) [15]. In SA, tammar wallabies have been introduced to four islands (Boston, Greenly, Granite, Wardang Islands) and in WA to North Island, Houtman Abrolhos Archipelago [15,16]. In the 1870s they were also liberated on Kawau Island, New Zealand and more recently around Rotorua on the North Island of New Zealand [18].

**Table 1:** Details of sampled island tammar wallaby (*Notamacropus eugenii*) populations [19–22].

	Island	Separation from mainland (yrs)	Area (ha)
Endemic			
	Kangaroo, SA	9 500	450 000
	Garden, WA	7 000	1 054
	Middle, WA	9 500	1 036
	West Wallabi, WA	11 500	587
	East Wallabi, WA	11 500	307
	North Twin Peak, WA	8 000	272
Introduced			
1983–5	North, WA		176
1870	Kawau, New Zealand		2 058

During the last 200 years, observed morphological differences amongst tammar wallaby populations has led to the description of 10 distinct species or subspecies (Table 2), although all are currently regarded as junior synonyms of *N*. *eugenii* [3,23]. However, the significant morphological differences, confirmed by subsequent analyses [15,24], are suggestive of genetic differentiation and divergence amongst allopatric populations although no comprehensive molecular genetic assessment has yet been made.

**Table 2:** Published scientific names applicable to the tammar wallaby (*Notamacropus eugenii*) [3,23].

Published name	Author and Year	Type Locality
*eugenii*	(Desmarest 1817)	St Peters Island, Nuyts, Archipelago, SA
*derbianus*	(Gray 1837)	WA
derbianus obscurior*	(Gray 1841)	Garden Island, WA
emiliae*	(Gray 1843)	Houtman Abrolhos, WA
*houtmanni*	(Gould 1844)	Houtman Abrolhos, WA
*dama*	(Gould 1844)	?
*gracilis*	(Gould 1844)	Lake Walyormouring, WA
*bedfordi*	(Thomas 1900)	?
*flindersi*	(Wood Jones 1924)	Flinders Island, Investigator Group, SA
*eugenii decres*	(Troughton 1941)	Kangaroo Island, SA

* *nomen nudum*

Previous genetic comparisons of the Kangaroo Island (SA) and Garden Island (WA) populations, using several techniques, revealed substantial differentiation (reviewed in [5], see also [25,26,27]), although captive mating trials demonstrated the complete fertility of F1 and back-cross hybrids of both sexes [28]. This combination of attributes was subsequently exploited [29] to enable the construction of the first comprehensive physical linkage map for a marsupial [30,31].

More recently, two microsatellite-based population genetic studies have compared tammar wallabies from Garden Island [32] and the Abrolhos Islands [22] with WA mainland populations, documenting significant genetic differentiation and reduced diversity within the island populations. In addition, the source of the introduced North Island population was identified as West Wallabi Island [29].

Microsatellite loci have also been used to compare the introduced New Zealand tammar wallaby populations (Kawau Island and Rotorua) with Kangaroo Island [21]. This study confirmed that, within New Zealand, the Rotorua population was sourced from Kawau Island. In addition, the Kawau Island population was considered unlikely to have been derived from Kangaroo Island, being thought more likely to represent the ‘extinct’ SA mainland population [21]. As a consequence, the trial reintroduction of tammar wallabies, sourced from Kawau Island, to Innes National Park on the SA mainland is underway [33].

The publication of the tammar wallaby genome [13] has reinforced this species’ role as a key model marsupial and it is increasingly used in genetic, genomic and other studies [e.g., 1,27,34,35–38]. However, to maximise its usefulness as a model organism there is also a need to assess the overall distribution of genetic diversity within the tammar wallaby and the degree of differentiation amongst populations. Since previous genetic studies have focused on different population subsets and used largely non-overlapping panels of neutral genetic markers, we have sampled all surviving endemic tammar wallaby populations and screened them for neutral diversity with a consistent set of autosomal and Y-linked microsatellite loci, as well as examining DNA sequence variation in the mitochondrial DNA (mtDNA) control region (*CR*). This will, for the first time, enable a comprehensive picture of the distribution of diversity within and amongst extant tammar wallaby populations to be compiled.

## Methods

### Sample collection and DNA extraction

In 2009 animals from Middle (n = 12) and North Twin Peak (n = 2) Islands were captured in cage traps as previously described [22]. A 2–5 mm diameter ear biopsy was collected from each and stored in 80% ethanol prior to DNA extraction using a standard high salt protocol [39]. Sample collection was undertaken in accordance with WA Department of Conservation permit 03/2009, and University of NSW Animal Ethics approval 06/103A. DNA samples from previous studies [21,22,32,40] were already available from seven populations—East Wallabi, West Wallabi and North Islands (n = 101) sampled 2006–2008; Kangaroo Island (n = 40) sampled 2003; Garden Island (n = 30) sampled 2000–2001; Tutanning (n = 63) sampled 2000–2001; Kawau Island (n = 30) sampled 1996. In addition, DNA samples collected between 1992 and 1996 from Perup (n = 6) and Middle Island (n = 5) Island (n = 3) were donated by other researchers.

### Microsatellite amplification and screening

Individuals were genotyped at 16 polymorphic autosomal microsatellite loci derived from the tammar wallaby (T3.1T, T15.1, T31.1, T32.1, T46.5, Me1, Me2, Me14, Me15, Me16, Me17 and Me28) and eastern grey kangaroo (*Macropus giganteus*) (G16.1, G20.2, G26.4, G31.1) [41–43], as previously described [44]. The genotypes for 252 individual *N*. *eugenii* are given in S1 Table. Males were also genotyped at four tammar wallaby derived Y-linked microsatellite loci (MeY01, MeY28, MeY37A, MeY37B) [45] as previously described [22]. PCR products were analysed on a AB 3730 DNA Analyser (Applied Biosystems, USA) at the Ramaciotti Centre, UNSW (9 loci) or at AGRF Melbourne (7 loci), with the resultant DNA fragments sized using GeneMapper v3.7 (Applied Biosystems, USA).

### Mitochondrial DNA amplification and screening

DNA sequence variation in the hypervariable Domain I of the mtDNA *CR* was determined using marsupial-specific primers [46] and single strand conformation polymorphism (SSCP [47]) as previously described [48]. All unique haplotypes were sequenced including multiple representations (up to four where available) using BigDye termination (Perkin-Elmer Applied Biosystems, Norwalk, CT, USA) and resolved in an AB 3730 DNA Analyser (Applied Biosystems, USA) at the Ramaciotti Centre, UNSW or on an ABI 377 Sequencer at Macquarie University. Sequences were edited using Sequencher v4.8 (Gene Codes Corporation, Ann Arbor, USA) and aligned using Clustal X [49].

### Estimates of microsatellite diversity

For the autosomal loci, conformance to Hardy-Weinberg equilibrium was conducted using GenePop v7 [50] via a Markov chain method (5000 iterations). The statistical significance levels were corrected for multiple comparisons using sequential Bonferroni adjustments [51]. Observed heterozygosity (*H*_*o*_), expected heterozygosity (*H*_*E*_), allelic diversity (*A*) and effective allelic diversity (*Ae*: corrected for sample size *n* = 15) were estimated using Fstat v2.9.3 [52]. The mean number of rare alleles (*rA*) (allele frequency ≤ 0.05) and unique (private) alleles (*uA*) per locus was also calculated. Differences in diversity indices amongst sampled populations were assessed via a Wilcoxon rank sign test using SYSTAT9. The effective inbreeding coefficient (*F*_*e*_, Wright’s fixation index) was calculated from the equation: *F*_*e*_ = 1 –*H*_*IS*_ / *H*_*M*_ where *H*_*IS*_ represents heterozygosity for island populations and *H*_*M*_ represents heterozygosity for mainland populations [53].

For Y-linked microsatellites the average number of alleles per locus (*A*) and haplotypic diversity (*h*) were calculated using GenAlEx v6.5 [54,55]. Allelic richness (*A*_*R*_) was calculated in FSTAT v2.9.3.

### Population differentiation

For the autosomal microsatellite data, several methods were used to infer population structure. Firstly, differentiation amongst populations was assessed by calculating pairwise *F*_*ST*_ [56], using FSTAT v.2.9.3, with significance tested via 900 permutations. Secondly, population genetic structure was also inferred using a Bayesian model-based clustering analysis in the program Structure v2.3.4 [57]. Structure was run with no *a priori* information on population assignment, under the admixture model with alpha inferred from the data, allele frequencies uncorrelated and lambda set to 1.0. After a burn-in of 200 000, 1 000 000 iterations were performed. For the whole data set, we tested the number of genetic clusters (populations, *K*) present using values of *K* between 1 and 12 with 10 replicates of each. The inferred number of populations within the sample was deduced using both maximum posterior probability (L(K) [57]), and maximum delta log likelihood (ΔK [58]) implemented in Structure Harvester 0.6.93 [59]. The resulting barplots were created in Distruct v1.1 [60]. Thirdly, principal component analysis (PCA) was used to assess the degree of genetic similarity amongst individuals, based on allele frequencies, using GenAlEx v6.5.

For the Y-linked microsatellite loci pairwise *ϕ*_*PT*_ values amongst populations were calculated using GenAlEx v6.5, with significance estimated using 999 permutations. A Y-haplotype network was also constructed in NETWORK v4.6.1.3 [61], using the TCS algorithm and the MP options to identify and remove unnecessary median vectors and links [62]. Loci with lower expected mutation rates were assigned higher weights, following [23], with perfect dinucleotide repeats (MeY01) weighted *w* = 2 and imperfect repeats (MeY28, MeY37A and MeY37B) weighted *w* = 5. For the mtDNA *CR* data pairwise differentiation (*Ф*_*ST*_) amongst populations was estimated and tested for significance in Arlequin v3.5 [63] using 5000 permutations.

### Phylogenetic analysis

Phylogenetic relationships amongst identified *N*. *eugenii CR* haplotypes were analysed using three methods (maximum parsimony (MP), maximum likelihood (ML), neighbour-joining (NJ)) in Paup* v4b10 [64]. MP analysis was undertaken using a Branch and Bound search, with furthest addition, gaps treated as a fifth state and 1 000 bootstrap replicates; for ML analysis a heuristic search was conducted under the HKY85+G+I model (selected using Modeltest v3.7 [65]), with random addition, TBR branch-swapping and 500 bootstrap replicates; NJ analysis was conducted using Kimura two-parameter model distances and 10 000 bootstrap replicates. Homologous *CR* sequences from an eastern grey kangaroo (*Macropus giganteus*) and western grey kangaroo (*M*. *fuliginosus*) (GenBank Accession numbers: AF443160 and AF443174, respectively), were used as outgroup taxa. For complete sequences of all new *CR* haplotypes see GenBank accession numbers KY623685-KY623712.

## Results

### Sample collection

Samples were obtained from 287 tammar wallabies from ten populations throughout the current range, including from Kangaroo Island, SA (*n* = 40) and Kawau Island, New Zealand (*n* = 30). From WA two mainland (Tutanning, *n* = 63; Perup *n* = 6) and six island (Garden Island, *n* = 30; Middle Island *n* = 17; North Twin Peak Island, *n* = 2; East Wallabi Island, *n* = 35; West Wallabi Island, *n* = 30; North Island, *n* = 36) populations were sampled.

### Microsatellite diversity

All of the 16 autosomal microsatellite loci were polymorphic in most populations (S2 Table). Exceptions were for nine loci on North Twin Peak Island (G20.2, G26.4, Me 2, Me14, Me1, Me16, Me17, Me28, T32.1), and three loci on each of Garden Island (G20.2, Me14, T15.1), Middle Island (Me16, Me17, T32.1) and North Island (G20.2, Me14, Me15). In all populations autosomal microsatellite genotype frequencies at all loci conformed to Hard-Weinberg expectations, except for Me16 in East Wallabi Island, G26.4 in Garden Island, T31.1 in North Twin Peak Island and G16.1, G20.2 and Me28 in Tutanning, where significant (*p*<0.05) deficiencies of heterozygotes were identified.

A total of 270 autosomal microsatellite alleles were identified; 174 in SA (33% unique) and 209 in WA (46% unique) (S2 Table). Of the unique SA alleles, 69% were larger than any of the WA alleles found at the same locus. Populations contained between 1–29 unique alleles, except for North and North Twin Peak Islands which shared all alleles with other populations. The sampled Kangaroo Island population contained 169 alleles (17% unique), while 85 alleles (2% unique) were detected in the Kawau Island (NZ) population. Most Kawau Island alleles (95%) were also found in the Kangaroo Island population. In WA, the combined island populations contained a similar total number of alleles (159 vs 161) and unique alleles (15% vs 14%) to the mainland. The two WA mainland sites (Tutanning and Perup) shared 40% of their alleles.

The Tutanning and Kangaroo Island populations had similar levels of autosomal microsatellite diversity for all parameters, except that the frequency of *rA* was significantly (*p*<0.05) higher in the Kangaroo Island population (Table 3). Values of *A*, *Ae*, *Ho*, *He*, *uA*, *rA* were significantly (*p*<0.05) lower in all island populations compared to the Tutanning and Kangaroo Island populations (except for values of *uA* between Tutanning and both Garden and Middle Island populations). The introduced North Island population had significantly (*p*<0.05) lower values of *A* and *Ae* than all other populations (Table 3). The Kawau Island population had significantly (*p*<0.05) lower diversity values for all parameters than the Kangaroo Island population. The Perup population had significantly lower *A*, *He* and *rA* than Tutanning, although this most likely reflects the large difference in sample size (Table 3).

**Table 3:** Genetic diversity estimates (mean ± SE) from 16 microsatellite loci in ten sampled tammar wallaby (*Notamacropus eugenii*) populations.

Population	*N*	*P*	*nA*	*A*	*Ae*	*Ho*	*He*	*uA*	*rA*	*Fe*
Kangaroo Is.	36	1.0	169	10.6±0.9	8.4±0.6	0.74±0.03	0.78±0.02	1.8±0.36	4.8±0.6	-
Kawau Is.	30	1.0	85	5.3±0.4	4.9±0.3	0.64±0.03	0.64±0.03	0.13±0.06	1.3±0.3	0.17
Tutanning	30	1.0	142	8.9±0.6	7.8±0.5	0.70±0.04	0.77±0.03	1.1±0.24	3.3±0.5	-
Perup	6	1.0	85	5.3±0.7	-	0.77±0.09	0.71±0.04	0.37±0.22	0.0	-
Garden Is.	30	0.81	65	4.1±0.5	3.5±0.4	0.37±0.05	0.41±0.06	0.38±0.10	1.5±0.3	0.47
East Wallabi Is.	35	1.0	65	4.1±0.4	3.6±0.4	0.41±0.05	0.43±0.05	0.25±0.09	1.1±0.1	0.44
West Wallabi Is.	30	1.0	60	3.8±0.3	3.4±0.2	0.43±0.04	0.46±0.04	0.19±0.07	1.1±0.2	0.40
North Is.	36	0.88	42	2.6±0.2	2.4±0.2	0.33±0.04	0.35±0.04	0.00	0.7±0.2	0.55
Middle Is.	17	0.81	65	4.1±0.7	4.0±0.5	0.44±0.08	0.48±0.08	0.56±0.26	0.5±0.3	0.37
North Twin Peak Is.	2	0.44	25	1.6±0.6	-	0.22±0.22	0.20±0.17	0.00	0.0	0.77

*N*, sample size; *P*, proportion of polymorphic loci; *nA*, total number of alleles; *A*, allelic diversity; *Ae*, effective number of alleles (*n* = 15); *Ho*, observed heterozygosity; *He*, expected heterozygosity; *uA*, number of unique alleles; *rA*, number of rare alleles; *Fe* effective inbreeding. Values for the Perup and North Twin Peak Island populations should be treated with caution due to small sample size.

Diversity at Y-linked loci was lower than autosomal loci, as expected (Tables 3 and 4). Between two and 12 alleles per locus were identified over the four Y-linked loci genotyped in 178 male tammar wallabies from nine populations (S3 Table). A total of 27 alleles were identified (4–16 per population), 16 in the SA (81% unique) and 14 (86% unique) in the WA population (Table 4). Overall 70% of alleles were shared amongst populations, although unique alleles were present in the Kangaroo Island, Middle Island and Tutanning populations (Table 4, S3 Table). These alleles formed 32 Y-haplotypes, 88% of which were population specific (Tables 4 and 5, S3 Table). Haplotypes were shared only between North and West Wallabi Islands (YH28), as well as between Kangaroo and Kawau Islands (YH1, YH2, YH3) (Table 5). While most (6/9) populations contained ≤3 Y-haplotypes, 16 were identified within the Kangaroo Island population alone (Tables 4 and 5), most other island populations showed no or limited diversity (Table 4).

**Table 4:** Genetic diversity estimates (mean ± SE) from four Y-linked microsatellite loci in nine sampled tammar wallaby (*Notamacropus eugenii*) populations.

Population	*N*	*A*	*Ae*	*nA*	*uA*	*nH*	*uH*	*h*
Kangaroo Is.	33	4.00 (±1.59)	2.14 (±0.67)	16	8	16	13	0.38 (±0.19)
Kawau Is.	18	2.00 (±0.41)	1.61 (±0.36)	8	0	5	2	0.39 (±0.13)
Tutanning	39	2.00 (±0.71)	1.46 (±0.49)	8	1	6	6	0.23 (±0.24)
Perup	3	1.00	1.00	4	0	1	1	-
Garden Is.	19	1.00	1.00	4	0	1	1	-
East Wallabi Is.	20	1.00	1.00	4	0	1	1	-
West Wallabi Is.	16	1.25 (±0.25)	1.19 (±0.19)	5	1	2	1	0.08 (±0.08)
North Is.	19	1.00	1.00	4	1	1	0	-
Middle Is.	11	1.50 (±0.29)	1.24 (±0.14)	6	2	3	3	0.08 (±0.05)

*N*, sample size; *A*, average alleles per locus; *Ae*, allelic richness (*n* = 3); *nA*, total alleles; *uA*, total unique alleles; *nH*, total haplotypes; *uH*, total unique haplotypes; *h* = haplotypic diversity.

**Table 5:** Distribution and frequency of Y chromosome haplotypes identified in nine sampled tammar wallaby (*Notamacropus eugenii*) populations.

Y Haplotype	Population								
	KI	KwI	Tut	Per	GI	EWI	WWI	NI	MI
1	6	3							
2	4	8							
3	3	5							
4	5								
5	3								
6	2								
7	1								
8	1								
9	1								
10	1								
11	1								
12	1								
13	1								
14	1								
15	1								
16	1								
17		2							
18		1							
19			14						
20			14						
21			4						
22			3						
23			2						
24			2						
25				3					
26					19				
27						20			
28							13	19	
29							3		
30									9
31									1
32									1

KI = Kangaroo Island; KwI = Kawau Island, New Zealand; Tut = Tutanning; Per = Perup; GI = Garden Island; EWI = East Wallabi Island; WWI = West Wallabi Island; NI = North Island; MI = Middle Island.

### MtDNA control region diversity

A total of 28 *CR* haplotypes were identified amongst the 206 tammar wallabies sampled from ten populations (Table 6). Within the aligned block of 595 bp, 122 variable sites were identified, 102 of which were phylogenetically informative. All populations contained multiple haplotypes (up to six), except for North Twin Peak Island (Table 6). Almost all (93%) identified *CR* haplotypes were population specific. Haplotypes were shared only between North and West Wallabi Islands (H20), as well as between Kangaroo and Kawau Islands (H2) (Table 6).

**Table 6:** Distribution and frequency of the 28 mitochondrial DNA control region haplotypes identified in ten sampled tammar wallaby (*Notamacropus eugenii*) populations.

MtDNA Haplotype	Population									
	KI	KwI	Tut	Per	GI	EWI	WWI	NI	MI	NTP
1	12									
2	7	22								
3	6									
4	1									
5		1								
6			9							
7			6							
8			3							
9			1							
10			1							
11			1							
12				4						
13				2						
14					17					
15					3					
16						29				
17						3				
18						1				
19							10			
20							10	12		
21							5			
22							4			
23								18		
24									12	
25									2	
26									1	
27									1	
28										2

KI = Kangaroo Island; KwI = Kawau Island, New Zealand; Tut = Tutanning; Per = Perup; GI = Garden Island; EWI = East Wallabi Island; WWI = West Wallabi Island; NI = North Island; MI = Middle Island; NTP = North Twin Peak Island.

There was substantial sequence divergence between *CR* haplotypes from SA and WA (14.54 ± 0.7%; mean ± sd); although more modest divergence amongst haplotypes within each region: range of 0.17–3.5% within SA and 0.17–6.4% within WA. Mean divergence within the Kangaroo Island population (1.4 ± 1.4%) was greater than that found between the Kangaroo and Kawau Island populations (0.89 ± 1.2%). Within WA, mean sequence divergence amongst haplotypes within endemic island populations was low (range 0.4–1.0%), but higher mean divergence was evident between island and WA mainland haplotypes (range 3.3–4.9%). WA mainland haplotypes differed by 0.2–5.5%, with a mean of 3.8 ± 1.0% separating the two sampled populations.

### Population differentiation

Significant genetic differentiation (*F*_*ST*_ and *Φ*_*ST*_) was detected amongst all adequately sampled populations (Table 7). Values were lowest between the two mainland WA populations (*F*_*ST*_ = 0.074; *Φ*_*ST*_ = 0.25), North and West Wallabi Island populations (*F*_*ST*_ = 0.15; *Φ*_*ST*_ = 0.097), as well as between Kangaroo and Kawau Islands (*F*_*ST*_ = 0.097; *Φ*_*ST*_ = 0.18) (Table 7). The WA island populations were highly differentiated from the WA mainland populations (mean *F*_*ST*_ = 0.32; *Φ*_*ST*_ = 0.69) and each other (mean *F*_*ST*_ = 0.47; *Φ*_*ST*_ = 0.86) (Table 7). The SA and WA populations were also highly differentiated (mean *F*_*ST*_ = 0.34; *Φ*_*ST*_ = 0.97).

**Table 7:** Genetic differentiation amongst ten sampled tammar wallaby (*Notamacropus eugenii*) populations.

	**KI**	**KwI**	**Tut**	**Per**	**GI**	**EWI**	**WWI**	**NI**	**MI**	**NTP**
Kangaroo Is.	-	**0.182**	**0.907**	**0.945**	**0.976**	**0.985**	**0.972**	**0.980**	**0.973**	**0.971**
Kawau Is.	**0.097**	-	**0.918**	**0.969**	**0.992**	**0.998**	**0.986**	**0.993**	**0.992**	**1.000**
Tutanning	**0.167**	**0.244**	-	**0.246**	**0.726**	**0.799**	**0.743**	**0.771**	**0.541**	**0.171**
Perup	**0.164**	**0.257**	**0.074**	-	**0.816**	**0.923**	**0.853**	**0.892**	**0.698**	**0.362**
Garden Is.	**0.358**	**0.445**	**0.310**	**0.388**	-	**0.973**	**0.934**	**0.958**	**0.942**	**0.948**
East Wallabi Is.	**0.356**	**0.418**	**0.331**	**0.374**	**0.517**	-	**0.664**	**0.784**	**0.974**	**0.999**
West Wallabi Is.	**0.328**	**0.395**	**0.313**	**0.357**	**0.489**	**0.495**	-	**0.079**	**0.932**	**0.928**
North Is.	**0.400**	**0.462**	**0.387**	**0.453**	**0.542**	**0.551**	**0.150**	-	**0.959**	**0.967**
Middle Is.	**0.298**	**0.388**	**0.218**	**0.274**	**0.452**	**0.498**	**0.433**	**0.512**	-	**0.852**
North Twin Peak Is.	**0.307**	**0.401**	0.214	0.255	0.523	**0.557**	**0.507**	**0.597**	0.283	-

Pairwise *Φ*_*ST*_ values for mtDNA data above the diagonal and pairwise *F*_*ST*_ for microsatellite data below the diagonal. KI = Kangaroo Island; KwI = Kawau Island, New Zealand; Tut = Tutanning; Per = Perup; GI = Garden Island; EWI = East Wallabi Island; WWI = West Wallabi Island; NI = North Island; MI = Middle Island; NTP = North Twin Peak Island. Values in bold are significant (*P*<0.05). Values for the Perup and North Twin Peak Island populations should be treated with caution due to small sample size.

**Fig 2:**
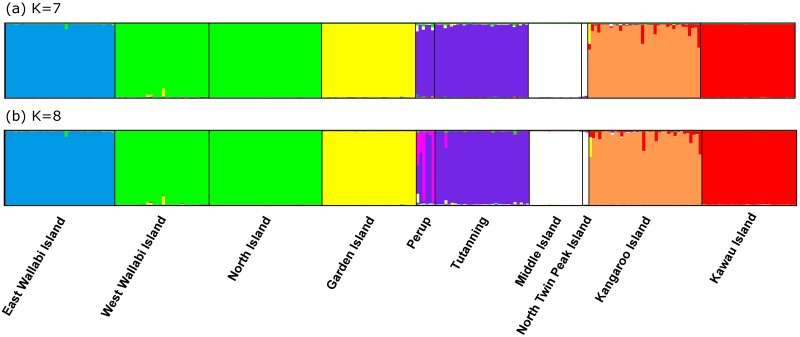
Structure plot (autosomal microsatellite loci) showing proportions of inferred ancestry (Q) in the K = 7 (a) and K = 8 (b) genetic clusters identified within tammar wallabies (*Notamacropus eugenii*) sampled from 10 sites.

The Bayesian model-based clustering analysis implemented in Structure indicated that either seven (maximum *L(K)*) or eight (maximum Δ*K*) populations were present in the data (S1 Fig). With K = 7, the inferred populations largely corresponded to sampling locations except for Perup and Tutanning, West Wallabi and North Islands, as well as Middle and North Twin Peak Islands where each pair was combined into single inferred populations (Fig 2a). With K = 8, the groupings were similar to K = 7 but with an additional cluster comprising some Perup individuals (Fig 2b).

**Fig 3:**
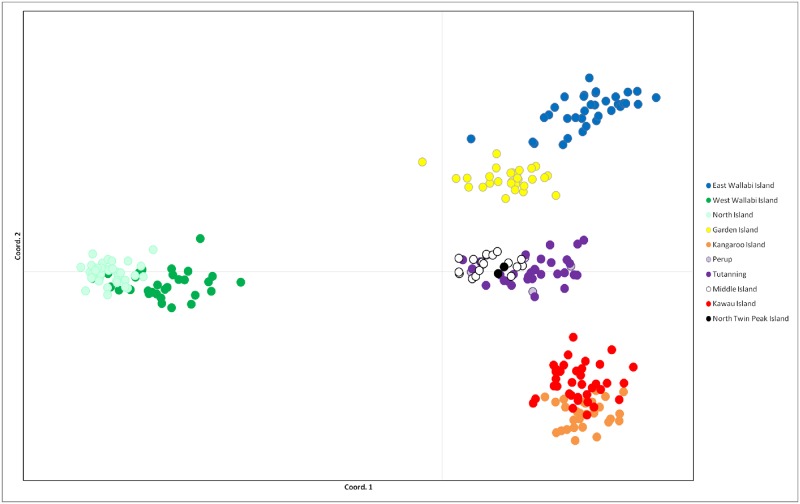
PCA plot (autosomal microsatellite data) showing four distinct genetic clusters of tammar wallabies (*Notamacropus eugenii*), corresponding to individuals sampled from East Wallabi and Garden Islands; West Wallabi and North Islands; Kangaroo and Kawau Islands; Perup, Tutanning, Middle Island and North Twin Peak Island.

The PCA of autosomal loci was plotted on two axes which cumulatively explained 55.32% of the variation (33.59 and 21.73% respectively) (Fig 3). The PCA plot revealed four main genetic clusters which represented samples from East Wallabi and Garden Islands, West Wallabi and North Islands, Kangaroo and Kawau Islands, and finally samples from Perup, Tutanning, Middle Island and North Twin Peak Island (Fig 3).

**Fig 4:**
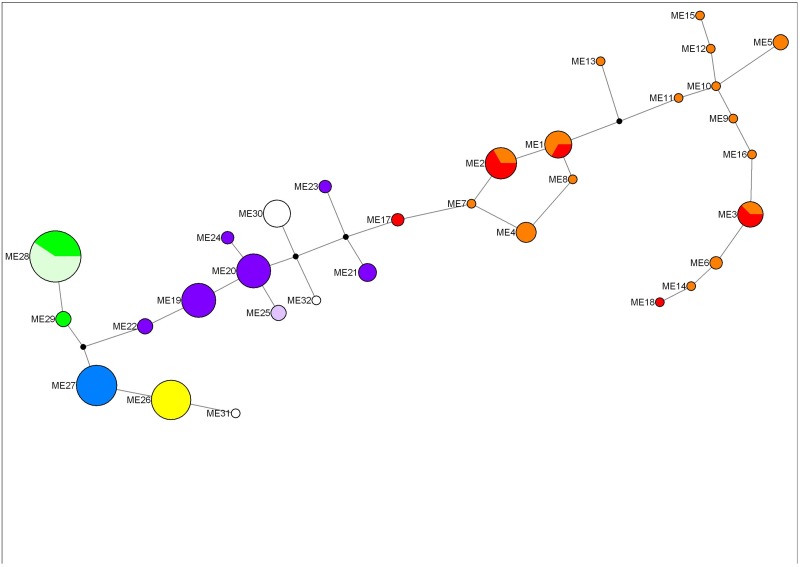
TCS network of Y-linked microsatellite haplotypes identified in nine tammar wallaby (*Notamacropus eugenii*) populations. Node size is proportional to haplotype frequency (Table 5). Black nodes are inferred intermediate haplotypes. Orange = Kangaroo Island; red = Kawau Island, New Zealand; dark purple = Tutanning; light purple = Perup; yellow = Garden Island; blue = East Wallabi Island; dark green = West Wallabi Island; light green = North Island; white = Middle Island.

The Y-linked microsatellite loci revealed a pattern of significant differentiation amongst all populations, except for North and West Wallabi Islands (*Φ*_*PT*_ = 0.15), and between Kangaroo and Kawau Islands (*Φ*_*PT*_ = 0.04) (Table 8). The WA island populations were highly differentiated from each other (mean *Φ*_*PT*_ = 0.86) and the WA mainland (mean *Φ*_*PT*_ = 0.82). The SA and WA populations were also well differentiated (mean *Φ*_*PT*_ = 0.72) (Table 8). An AMOVA revealed that genetic diversity was significantly partitioned between SA and WA populations (F_*CT*_ = 0.41, *P* = 0.03). Distinct eastern (SA) and western (WA) clusters were also apparent in the Y haplotype network (Fig 4).

**Table 8:** Genetic differentiation, for Y-linked microsatellite data, amongst nine sampled tammar wallaby (*Notamacropus eugenii*) populations.

	**KI**	**KwI**	**Tut**	**Per**	**GI**	**EWI**	**WWI**	**NI**	**MI**
Kangaroo Is.	-	0.107	**0.001**	**0.012**	**0.001**	**0.001**	**0.001**	**0.001**	**0.001**
Kawau Is.	0.036	-	**0.001**	**0.021**	**0.001**	**0.001**	**0.001**	**0.001**	**0.001**
Tutanning	0.726	0.779	-	**0.001**	**0.001**	**0.001**	**0.001**	**0.001**	**0.001**
Perup	0.275	0.406	0.769	-	**0.001**	**0.001**	**0.001**	**0.001**	**0.001**
Garden Is.	0.780	0.865	0.579	1.000	-	**0.001**	**0.001**	**0.001**	**0.001**
East Wallabi Is.	0.784	0.869	0.781	1.000	1.000	-	**0.001**	**0.001**	**0.001**
West Wallabi Is.	0.742	0.816	0.659	0.930	0.926	0.928	-	0.085	**0.001**
North Is.	0.780	0.865	0.725	1.000	1.000	1.000	0.153	-	
Middle Is.	0.685	0.765	0.646	0.904	0.930	0.929	0.829	0.934	-

Pairwise *Φ*_*PT*_ values below diagonal and significance (based on 999 permutations) above diagonal. KI = Kangaroo Island; KwI = Kawau Island, New Zealand; Tut = Tutanning; Per = Perup; GI = Garden Island; EWI = East Wallabi Island; WWI = West Wallabi Island; NI = North Island; MI = Middle Island. Values in bold are significant (*P*<0.05).

### Phylogenetic analysis

**Fig 5:**
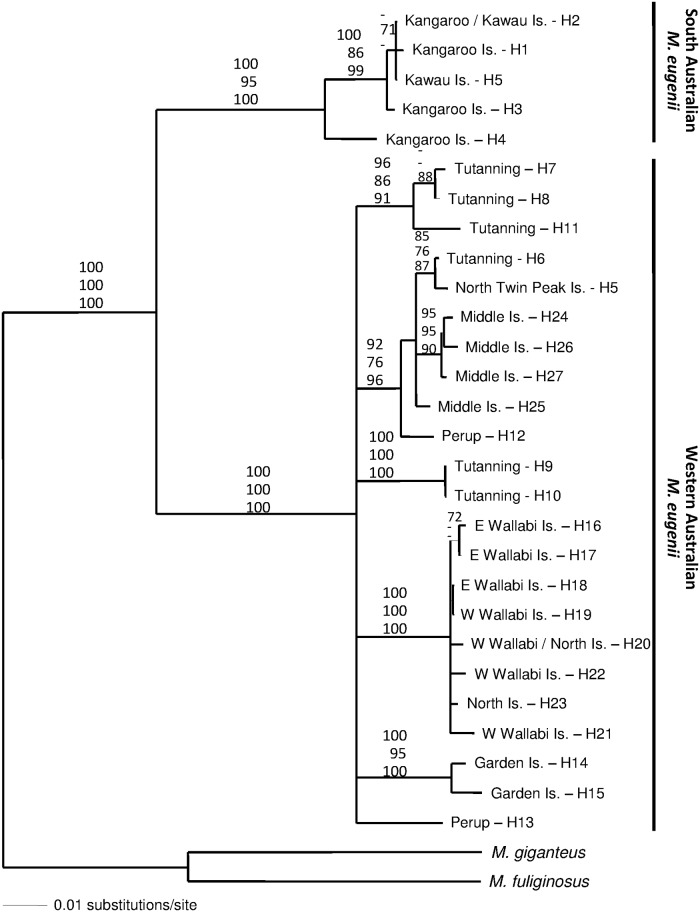
Phylogenetic relationships amongst mtDNA *CR* haplotypes identified from ten tammar wallaby (*Notamacropus eugenii)* populations from South Australia, Western Australia and New Zealand. Haplotypes from *Macropus giganteus* and *M*. *fuliginosus* were used as outgroups. Numbers on branches indicate percent of bootstrap replicates when ≥ 70% (maximum parsimony, maximum likelihood, neighbour-joining).

Phylogenetic analyses of the tammar wallaby mtDNA *CR* revealed two well supported clusters of highly divergent haplotypes, showing complete reciprocal monophyly and corresponding to the sampled SA and WA populations (Fig 5). Within the WA clade, each island population tended to have monophyletic clusters of unique haplotypes. However, the relationship amongst these island lineages and between them and the WA mainland haplotypes was largely unresolved. Within the SA clade there was no phylogenetic separation between the identified Kangaroo and Kawau Island haplotypes (Fig 5).

## Discussion

### Differentiation between WA and SA

Both our mtDNA and microsatellite analyses have revealed significant differentiation amongst *N*. *eugenii* populations throughout the species’ range. Substantial divergence was identified between eastern (SA) and western (WA) populations, with each region being characterised by a high proportion of unique and divergent alleles/haplotypes. This pattern of divergence is consistent with the long-term isolation of SA and WA *N*. *eugenii* populations and the lack of recent gene flow between them, allowing each to evolve independently. A similar pattern of genetic divergence is also present between eastern and western populations (or species pairs) in many taxa across southern Australia, including grey kangaroos [66], dasyurids [67,68], bandicoots [69], rodents [70], birds [71–73] and reptiles [74]. This widespread pattern of east/west divergence is believed to reflect the separation of mesic fauna by the arid Nullarbor Barrier, as a consequence of the increasing aridity of Australia during the Pleistocene [75,76]. Although [77] suggested tammar wallabies in WA and SA were separated over 30 000–50 000 years ago, the substantial genetic differentiation detected in our study, especially for mtDNA, indicates a much older divergence ~1MYA [78].

The divergence in *CR* sequence (14.5%) detected here between eastern and western tammar wallabies is similar to that reported between eastern and western grey kangaroos (14%) [66], and amongst six species of rock-wallabies (*Petrogale* spp. 10–17% [79,80]). In addition, differences in allozymes between the Kangaroo Is. (SA) and Garden Is. (WA) populations are similar to that typically found between species [5,15]. Morphological analysis has also revealed two main clusters within *N*. *eugenii*, largely reflecting distinct eastern and western groupings, although with alterative clustering for some southern island populations [15,24].

Since the allopatric populations of SA and WA tammar wallabies are genetically highly differentiated, with levels of divergence typical of different species, there would be some justification in recognising each as a separate species, for consistency with data from other macropodid species. However, despite their genetic divergence at neutral loci, SA and WA tammar wallabies are known to be fully inter-fertile (in captivity), with F1 and back-cross hybrids of both sexes showing normal fertility [28]. This is quite unlike the similarly divergent eastern and western grey kangaroos, where both pre- and post-mating reproductive isolation is more developed, including male hybrid sterility [81,82]. In addition, eastern and western grey kangaroos occur in sympatry across large areas of eastern Australia with introgression being only occasionally detected [66]. In contrast, SA and WA tammar wallabies are naturally allopatric, preventing a direct test of reproductive isolation under field conditions. Since SA and WA tammar wallabies have been shown to be potentially interbreeding, at least in captivity, the available evidence is consistent with them constituting a single species as defined by the Biological Species Concept [83]. Therefore, we recommend that a single species of tammar wallaby (*N*. *eugenii*) continue to be recognised. There is however, the need for further research, since a reduced breeding efficiency when producing Garden Island by Kangaroo Island F1 hybrids in captivity has been reported [24]. It is therefore possible that some incipient pre- or post-mating reproductive isolation is present. This needs to be more thoroughly assessed, since the reported reduced breeding efficiency [24] may be related to the lower reproductive rate observed in Garden Island tammar wallabies under captive conditions (in eastern Australia). Additional experiments should therefore be conducted to assess behavioural interactions and simultaneous mate-choice under more natural conditions, as well as the capacity of Kangaroo Island tammar wallabies to hybridise with individuals from other WA populations. These observations also have implications for conservation biology and taxonomy as they demonstrate that allopatric populations differentiated at neutral loci are not necessarily reproductively isolated, since reproductive isolation appears more associated with differential environmental adaptation rather than geographic isolation and drift [84,85].

The genetic divergence detected between WA and SA tammar populations is sufficient for them to be recognised as separate Evolutionarily Significant Units (ESUs) (*sensu* [86]). We would suggest that the divergence recognised by ESUs is often equivalent to the concept of subspecies, although accepted criteria to define subspecies remain elusive and controversial [87,90]. Nevertheless, we believe they can play a useful role in identifying major geographically, genetically and/or morphologically distinct subpopulations within species and so we suggest that eastern *N*. *eugenii* populations (SA) be known as *N*. *eugenii eugenii*, and western (WA) populations as *N*. *eugenii derbianus* (Table 2) as recently proposed [3]. This arrangement assumes that the tammar wallabies from the type locality (St Peter Island, Nuyts Archipelago, SA), which are extinct and were not examined in this study, group with the sampled SA populations. Material from the type locality was also not included in two studies of tammar wallaby cranial morphometrics [15,24], although skulls from Flinders Island, Investigator Group, SA (located south-east of St Peter Is.) were examined. While one study [15] concluded that the Flinders Island tammar wallabies were most similar to those from the southwest WA mainland, another [20] concluded they grouped with the SA mainland and Kangaroo Island populations. Therefore, until the relationship of the St Peter Island tammar wallabies can be directly clarified, perhaps using ancient DNA from the very limited skeletal material present in museums, it will remain somewhat uncertain whether the name *eugenii* correctly applies to the eastern or western tammar wallaby populations.

The western affinity of some SA animal populations is not unprecedented; for example, the Pearson Island rock-wallaby (*Petrogale lateralis pearsoni*) also found in the Investigator Group, SA is most closely related to the black-footed rock-wallaby (*P*. *l*. *lateralis*) from southwest WA [79]. Similarly, a number of largely south-western WA bird, reptile and mammal species reach their eastern limit on the Eyre Peninsula of SA (eg, little long-tailed dunnart, western yellow robin, rufous tree-creeper [88,89]). However, recent molecular studies of Australian tiger snakes (*Notechis scutatus*) [74] and southern brown bandicoots (*Isoodon obesulus*) [69] from the Nuyts Archipelago have shown their affinities lie with south-eastern rather than south-western populations.

### Differentiation within WA

Although we recommend that a single subspecies be recognised in WA we note that substantial differentiation in microsatellite loci also occurs among many of the sampled WA populations, some of which have historically been proposed as separate taxa (Table 2). However, the level of mtDNA divergence amongst WA populations appears insufficient to warrant the recognition of further subspecies. For example, the mean *CR* sequence divergence between WA island and mainland populations ranged from 3.3–4.9%, but up to 5.5% divergence was found between haplotypes within the Tutanning population alone. Similarly, although the East and West Wallabi Islands populations appear highly divergent based on autosomal microsatellite data (Table 7, Fig 4) they have almost identical CR haplotypes (Fig 5) indicating very recent common ancestry. Thus the genetically (Fig 4) and morphologically distinct WA island populations [15,24] are most likely the consequence of relatively recent divergence under the influence of small population size, genetic drift and adaptation to an island environment, following their isolation 7 000–11 500 years ago by rising sea levels (Table 1). These recent and relatively rapid evolutionary processes are also reflected in their genetic profiles, with each island having significantly reduced diversity and thus show exaggerated genetic differentiation from each other and the WA mainland populations [90]. While individually each island population is genetically depauperate and inbred (Table 3), together they preserve considerable diversity and also retain unique alleles and haplotypes. As such, these WA island populations represent a valuable genetic resource and have high conservation value, a situation similar to that reported for WA populations of the northern quoll (*Dasyurus hallucatus*) [91].

An exception is the North Twin Peak Island tammar wallaby population, which was found to share all of its autosomal microsatellite alleles with the nearby (31 km west) Middle Island population. Whether this similarity reflects recent gene flow or the preferential retention in both populations of the higher frequency alleles present in the common ancestral population, must await more comprehensive sampling of the North Twin Peak population (currently *n* = 2). However, since the populations do not share *CR* haplotypes and are morphologically distinct (unpublished data) the later hypothesis seems more likely.

On the WA mainland some genetic differentiation is also apparent between the two sampled southwest WA populations (Tutanning and Perup). Although only a limited sample was available from Perup (*n* = 6), unique *CR* and Y haplotypes were detected and 55% of autosomal microsatellite alleles were not shared with the much better sampled Tutanning (*n* = 50) population located 200 km to the northeast. These preliminary data suggest that mainland WA populations are also structured with limited gene flow by both sexes between sites. More comprehensive sampling of remaining tammar wallaby populations throughout southwest WA is required to confirm these findings. However, if these data are typical then considerable unique diversity may exist within each remaining mainland WA population. In this context, it would also be important to examine the pattern of male and female mediated gene flow and extent of population genetic structure throughout the abundant Kangaroo Island tammar population. Since Kangaroo Island is over 150 km long, tammar wallaby populations may show significant genetic structure across the island.

### Differentiation and diversity within SA populations

Although the Kangaroo Island and Kawau Island populations showed some differentiation in several analyses they were more similar than expected, given the latter is thought to represent the SA mainland population and they have been considered distinct subspecies [21,33,92]. The Kawau Island population shared most microsatellite alleles, as well as Y and *CR* haplotypes with the Kangaroo Island population. This lack of substantial differentiation was in contrast to all other island-mainland population comparisons and was more similar to the West Wallabi / North Island comparison. However, the shallow divergence between the Kangaroo Island and Kawau Island populations does not necessarily undermine the case for a SA mainland origin of the Kawau Island population [21]. Since Kangaroo Island is a large island (450 000 ha), supporting a substantial tammar population (up to 106 [93]) that became isolated relatively recently (~9500 ybp), the impact of genetic drift in promoting genetic divergence from the mainland is likely to be much slower than for the considerably smaller WA islands (all < ~1000 ha; Table 1) examined. There is also the possibility that allele and haplotype frequencies in Kawau Island were distorted during the establishment of this population in New Zealand, from a small number of founders, so that unique and rare alleles were preferentially lost. Although two unique Y haplotypes and one unique mtDNA *CR* haplotype were detected in the Kawau Island population these were all very similar (1 mutational step) to haplotypes recorded in Kangaroo Island and so may represent recent mutations in the Kawau Island population or be as yet unsampled in the Kangaroo Island population. In light of this uncertainty, definitive conclusions as to the origins of the Kawau Island population and the distinction of the SA mainland and Kangaroo Island populations remain elusive. To further resolve this matter would require not only genetic data from definitive historic SA mainland tammar wallabies (derived from museum material), but also a better understanding of the distribution of genetic diversity throughout Kangaroo Island, since the population we sampled is from the western end of the island and the degree of population structure across the island remains unknown. The similarity between the Kangaroo Island and Kawau Island tammar populations detected in this study should not impact the ongoing re-introduction of Kawau-derived tammar wallabies to the Yorke Peninsula on the mainland SA, since returning SA tammar wallabies to the mainland is a worthy endeavour for biodiversity conservation and restoring ecosystem function. However the recent use of *eugenii eugenii* to refer to the extinct SA mainland tammar wallaby population as distinct from *eugenii decres* on Kangaroo Island (e.g. [33,92]) is inappropriate; for if distinct island subspecies were to be recognised, *eugenii eugenii* would be most accurately associated with the extinct St Peters Island population (type locality) and no scientific name has yet been specifically associated with the SA mainland population (Table 2).

Although island populations typically have reduced diversity compared to mainland populations [32,94], a remarkable feature of these data is the high genetic diversity detected in the Kangaroo Island tammar wallaby population. For autosomal microsatellites the levels of diversity (*A*, *He*) are amongst the highest yet reported in marsupials [2]. A remarkably high number of Y haplotypes were also detected in the Kangaroo Island population (Table 4, [27]), compared to other tammar populations, a more widespread and abundant macropodid (i.e. western grey kangaroo [95]) and many other species which typically show low variation at sex chromosome loci [96]. These high levels of diversity may be a consequence of Kangaroo Island’s large size (Table 1), which has enabled the tammar wallabies to retain a large *Ne* since isolation from the mainland population and so reduce the impact of genetic drift [94,97]. Some macropodid populations on other large Australian islands, for example King and Flinders Islands, also show high diversity [98,99], although not the sympatric western grey kangaroo population on Kangaroo Island [78,100]. The now extinct SA mainland tammar wallaby population is therefore likely to have also been highly diverse, maybe even more so than surviving mainland populations in WA. Reduced diversity in WA populations is hypothesised from biogeography, since tammar wallabies are thought to have spread from eastern to western Australia across the arid Nullabor Barrier [77]. Similarly, in the western grey kangaroo, an expansion across southern Australia (although in the opposite direction) resulted in reduced genetic diversity in the more recently colonised population [78,95,100]. However, determining the original levels of diversity in SA and WA mainland tammar wallaby populations prior to their recent decline, and in SA extinction, is now almost impossible due to poor historic sampling.

### Conclusions

To date, most studies of tammar wallaby physiology, reproduction, genetics and development have utilised the Kangaroo Island population, and they are now amongst the best known of marsupials [4,11]. We hope that the significant genetic divergence between SA and WA tammar wallabies revealed in this study will now encourage similar detailed investigations of the diverse WA populations, as their relatively long isolation from the well-researched SA population, their larger latitudinal range and greater diversity in body size, salt and 1080 tolerance and habitat [4] is likely to have resulted in the development of alternate strategies and metabolic pathways. For example, the Kangaroo Island tammar wallaby is well known for its highly synchronised breeding linked to the summer solstice [10]. This is one of only two macropodid species that employ both strict seasonal and lactational control of reproductive quiescence. However, the other species, red-necked wallaby (*Notamacropus rufogriseus*), employs different strategies at different latitudes [10]. The extent to which the control of reproduction varies in tammar wallaby populations across their latitudinal range should also be investigated. Access to the tammar genome [13] and advances in Next Generation sequencing technologies will greatly facilitate the identification, characterisation and utility of variant traits in this model organism, which in turn will add significantly to our understanding of macropodid and marsupial evolutionary biology.

## Supporting information

S1 Table Microsatellite genotypes at 16 autosomal loci in ten tammar wallaby (*Notamacropus eugenii*) populations. (XLSX)

S2 Table Allele frequencies for 16 autosomal microsatellite loci in ten tammar wallaby (*Notamacropus eugenii*) populations. KI = Kangaroo Island; KwI = Kawau Island, New Zealand; Tut = Tutanning; Per = Perup; GI = Garden Island; EWI = East Wallabi Island; WWI = West Wallabi Island; NI = North Island; MI = Middle Island; NTP = North Twin Peak Island.
(DOCX)

S3 Table Allelic combinations of the 32 Y haplotypes identified in nine tammar wallaby (*Notamacropus eugenii*) populations. KI = Kangaroo Island; KwI = Kawau Island, New Zealand; Tut = Tutanning; Per = Perup; GI = Garden Island; EWI = East Wallabi Island; WWI = West Wallabi Island; NI = North Island; MI = Middle Island.
(DOCX)

S1 Fig Structure output showing a) maximum *L(K)* at K = 7 and b) maximum Δ*K* at K = 8 (b). (DOCX)
