# Supplementary material for: Extensive genetic differentiation detected within a model marsupial, the tammar wallaby (Notamacropus eugenii)
Source: PLoS One. 2017 Mar 3;12(3):e0172777. doi: 10.1371/journal.pone.0172777 (PMC5336229; doi:10.1371/journal.pone.0172777)
Supplement: S2 Table — KI = Kangaroo Island; KwI = Kawau Island, New Zealand; Tut = Tutanning; Per = Perup; GI = Garden Island; EWI = East Wallabi Island; WWI = West Wallabi Island; NI = North Island; MI = Middle Island; NTP = North Twin Peak Island. (DOCX) [file pone.0172777.s002.docx]

### S1 Table. Allele frequencies for 16 autosomal microsatellite loci in ten tammar wallaby (*Notamacropus eugenii*) populations.

| **Locus** | | **Allele** | | **KI** | | **KwI** | | **Tut** | | **Per** | | **GI** | | **EWI** | | **WWI** | | **NI** | | **MI** | **NTP** |
| --- | --- | --- | --- | --- | --- | --- | --- | --- | --- | --- | --- | --- | --- | --- | --- | --- | --- | --- | --- | --- | --- |
| Me2 | | 223 | |  | |  | |  | |  | | 0.217 | |  | |  | |  | |  |  |
|  | | 227 | |  | |  | |  | |  | |  | |  | |  | |  | | 0.059 |  |
|  | | 229 | |  | |  | | 0.017 | |  | |  | |  | |  | |  | |  |  |
|  | | 231 | |  | |  | | 0.133 | | 0.500 | |  | |  | |  | |  | |  |  |
|  | | 233 | | 0.014 | |  | |  | |  | | 0.767 | |  | |  | |  | |  |  |
|  | | 235 | |  | |  | | 0.083 | |  | | 0.017 | |  | |  | |  | |  |  |
|  | | 237 | | 0.125 | |  | | 0.067 | | 0.250 | |  | |  | |  | |  | | 0.059 |  |
|  | | 239 | |  | |  | | 0.200 | |  | |  | |  | |  | |  | |  |  |
|  | | 241 | |  | |  | | 0.033 | |  | |  | |  | |  | |  | | 0.324 |  |
|  | | 243 | | 0.069 | |  | | 0.083 | | 0.083 | |  | |  | |  | |  | | 0.559 | 1.000 |
|  | | 247 | | 0.069 | |  | | 0.017 | |  | |  | | 0.471 | |  | |  | |  |  |
|  | | 249 | |  | |  | | 0.133 | |  | |  | | 0.397 | |  | |  | |  |  |
|  | | 251 | |  | |  | |  | |  | |  | | 0.118 | |  | |  | |  |  |
|  | | 253 | | 0.028 | |  | | 0.200 | | 0.083 | |  | |  | |  | |  | |  |  |
|  | | 255 | | 0.292 | | 0.567 | |  | |  | |  | |  | |  | |  | |  |  |
|  | | 257 | |  | |  | |  | |  | |  | |  | | 0.200 | | 0.014 | |  |  |
|  | | 259 | | 0.056 | |  | | 0.033 | |  | |  | | 0.015 | | 0.383 | | 0.931 | |  |  |
|  | | 261 | | 0.208 | | 0.083 | |  | |  | |  | |  | | 0.217 | | 0.042 | |  |  |
|  | | 263 | | 0.097 | | 0.133 | |  | |  | |  | |  | | 0.200 | | 0.014 | |  |  |
|  | | 265 | | 0.028 | | 0.183 | |  | | 0.083 | |  | |  | |  | |  | |  |  |
|  | | 267 | |  | | 0.033 | |  | |  | |  | |  | |  | |  | |  |  |
|  | | 269 | | 0.014 | |  | |  | |  | |  | |  | |  | |  | |  |  |
| Me14 | | 160 | |  | |  | | 0.083 | |  | | 1.000 | |  | |  | |  | |  |  |
|  | | 162 | | 0.014 | |  | |  | |  | |  | | 0.757 | |  | |  | |  |  |
|  | | 164 | |  | |  | |  | |  | |  | | 0.014 | | 0.117 | | 0.042 | |  |  |
|  | | 166 | |  | |  | | 0.117 | | 0.333 | |  | | 0.186 | | 0.283 | | 0.639 | | 0.029 |  |
|  | | 168 | |  | |  | | 0.367 | | 0.083 | |  | | 0.043 | | 0.467 | | 0.319 | | 0.618 | 1.000 |
|  | | 170 | |  | |  | | 0.133 | | 0.083 | |  | |  | | 0.017 | |  | | 0.353 |  |
|  | | 172 | |  | |  | | 0.067 | | 0.083 | |  | |  | | 0.117 | |  | |  |  |
|  | | 174 | |  | |  | | 0.133 | |  | |  | |  | |  | |  | |  |  |
|  | | 176 | | 0.111 | | 0.150 | | 0.100 | |  | |  | |  | |  | |  | |  |  |
|  | | 178 | | 0.153 | | 0.417 | |  | |  | |  | |  | |  | |  | |  |  |
|  | | 180 | | 0.069 | | 0.167 | |  | |  | |  | |  | |  | |  | |  |  |
|  | | 182 | | 0.139 | |  | |  | | 0.083 | |  | |  | |  | |  | |  |  |
|  | | 184 | | 0.069 | | 0.050 | |  | | 0.333 | |  | |  | |  | |  | |  |  |
|  | | 186 | | 0.014 | | 0.017 | |  | |  | |  | |  | |  | |  | |  |  |
|  | | 188 | | 0.069 | |  | |  | |  | |  | |  | |  | |  | |  |  |
|  | | 190 | | 0.083 | |  | |  | |  | |  | |  | |  | |  | |  |  |
|  | | 192 | | 0.083 | | 0.117 | |  | |  | |  | |  | |  | |  | |  |  |
|  | | 194 | | 0.056 | |  | |  | |  | |  | |  | |  | |  | |  |  |
|  | | 196 | | 0.014 | |  | |  | |  | |  | |  | |  | |  | |  |  |
|  | | 198 | | 0.056 | | 0.067 | |  | |  | |  | |  | |  | |  | |  |  |
|  | | 200 | | 0.028 | | 0.017 | |  | |  | |  | |  | |  | |  | |  |  |
|  | | 202 | | 0.042 | |  | |  | |  | |  | |  | |  | |  | |  |  |
| G16-1 | | 142 | |  | |  | |  | | 0.083 | |  | |  | |  | |  | |  |  |
|  | | 150 | |  | |  | |  | |  | |  | |  | |  | |  | | 0.118 |  |
|  | | 152 | |  | |  | | 0.333 | | 0.167 | |  | |  | |  | |  | |  |  |
|  | | 154 | |  | |  | | 0.033 | | 0.417 | | 0.450 | |  | | 0.917 | | 0.971 | | 0.471 | 0.750 |
|  | | 156 | |  | | 0.033 | | 0.317 | | 0.250 | | 0.033 | |  | |  | |  | | 0.147 | 0.250 |
|  | | 158 | |  | |  | | 0.017 | | 0.083 | | 0.017 | | 0.957 | | 0.083 | | 0.014 | | 0.265 |  |
|  | | 160 | | 0.083 | |  | | 0.133 | |  | | 0.100 | | 0.043 | |  | | 0.014 | |  |  |
|  | | 162 | | 0.042 | |  | | 0.033 | |  | | 0.183 | |  | |  | |  | |  |  |
|  | | 164 | | 0.069 | | 0.100 | | 0.083 | |  | | 0.200 | |  | |  | |  | |  |  |
|  | | 166 | | 0.250 | | 0.217 | | 0.050 | |  | | 0.017 | |  | |  | |  | |  |  |
|  | | 168 | | 0.375 | | 0.433 | |  | |  | |  | |  | |  | |  | |  |  |
|  | | 170 | | 0.125 | | 0.083 | |  | |  | |  | |  | |  | |  | |  |  |
|  | | 172 | | 0.028 | | 0.133 | |  | |  | |  | |  | |  | |  | |  |  |
|  | | 174 | | 0.028 | |  | |  | |  | |  | |  | |  | |  | |  |  |
| G20-2 | | 133 | |  | |  | | 0.107 | | 0.083 | | 1.000 | | 0.914 | | 0.050 | |  | |  |  |
|  | | 135 | |  | |  | |  | | 0.083 | |  | |  | |  | |  | |  |  |
|  | | 137 | |  | |  | | 0.286 | | 0.167 | |  | |  | |  | |  | |  |  |
|  | | 139 | |  | |  | | 0.375 | | 0.083 | |  | | 0.086 | |  | |  | |  |  |
|  | | 141 | | 0.044 | |  | | 0.125 | | 0.417 | |  | |  | |  | |  | | 0.206 |  |
|  | | 143 | | 0.015 | |  | | 0.036 | | 0.083 | |  | |  | | 0.117 | |  | | 0.588 | 1.000 |
|  | | 145 | |  | |  | | 0.036 | | 0.083 | |  | |  | | 0.817 | | 1.000 | | 0.206 |  |
|  | | 149 | | 0.044 | |  | |  | |  | |  | |  | |  | |  | |  |  |
|  | | 151 | | 0.176 | | 0.033 | | 0.018 | |  | |  | |  | | 0.017 | |  | |  |  |
|  | | 153 | | 0.015 | | 0.200 | |  | |  | |  | |  | |  | |  | |  |  |
|  | | 155 | | 0.029 | | 0.150 | | 0.018 | |  | |  | |  | |  | |  | |  |  |
|  | | 157 | | 0.029 | |  | |  | |  | |  | |  | |  | |  | |  |  |
|  | | 159 | | 0.029 | |  | |  | |  | |  | |  | |  | |  | |  |  |
|  | | 161 | | 0.103 | |  | |  | |  | |  | |  | |  | |  | |  |  |
|  | | 163 | | 0.162 | | 0.250 | |  | |  | |  | |  | |  | |  | |  |  |
|  | | 165 | | 0.235 | | 0.033 | |  | |  | |  | |  | |  | |  | |  |  |
|  | | 167 | | 0.059 | | 0.100 | |  | |  | |  | |  | |  | |  | |  |  |
|  | | 169 | | 0.029 | | 0.233 | |  | |  | |  | |  | |  | |  | |  |  |
|  | | 171 | | 0.015 | |  | |  | |  | |  | |  | |  | |  | |  |  |
|  | | 173 | | 0.015 | |  | |  | |  | |  | |  | |  | |  | |  |  |
| G26-4 | | 171 | |  | |  | |  | | 0.167 | |  | |  | |  | |  | |  |  |
|  | | 175 | | 0.014 | |  | | 0.950 | | 0.667 | | 0.733 | |  | |  | |  | | 0.941 | 1.000 |
|  | | 177 | |  | |  | |  | |  | |  | | 0.986 | | 0.650 | | 0.736 | |  |  |
|  | | 179 | |  | |  | |  | |  | |  | |  | |  | |  | | 0.059 |  |
|  | | 181 | |  | |  | | 0.050 | | 0.167 | | 0.267 | | 0.014 | | 0.350 | | 0.264 | |  |  |
|  | | 193 | | 0.028 | |  | |  | |  | |  | |  | |  | |  | |  |  |
|  | | 197 | |  | | 0.083 | |  | |  | |  | |  | |  | |  | |  |  |
|  | | 201 | | 0.153 | | 0.183 | |  | |  | |  | |  | |  | |  | |  |  |
|  | | 205 | | 0.111 | |  | |  | |  | |  | |  | |  | |  | |  |  |
|  | | 209 | | 0.222 | | 0.383 | |  | |  | |  | |  | |  | |  | |  |  |
|  | | 211 | | 0.056 | |  | |  | |  | |  | |  | |  | |  | |  |  |
|  | | 213 | | 0.236 | | 0.117 | |  | |  | |  | |  | |  | |  | |  |  |
|  | | 217 | | 0.139 | |  | |  | |  | |  | |  | |  | |  | |  |  |
|  | | 219 | | 0.028 | | 0.150 | |  | |  | |  | |  | |  | |  | |  |  |
|  | | 223 | | 0.014 | | 0.083 | |  | |  | |  | |  | |  | |  | |  |  |
| G31-1 | | 115 | |  | |  | |  | |  | | 0.017 | |  | |  | |  | |  |  |
|  | | 117 | | 0.028 | |  | |  | |  | | 0.817 | | 0.014 | | 0.567 | | 0.583 | |  |  |
|  | | 119 | |  | | 0.155 | | 0.083 | |  | |  | | 0.957 | |  | |  | |  |  |
|  | | 121 | | 0.486 | | 0.448 | |  | |  | |  | | 0.029 | |  | |  | |  |  |
|  | | 123 | | 0.347 | | 0.379 | | 0.183 | | 0.083 | |  | |  | |  | |  | |  |  |
|  | | 125 | | 0.139 | | 0.017 | | 0.233 | | 0.333 | | 0.017 | |  | |  | |  | | 0.235 | 0.250 |
|  | | 127 | |  | |  | | 0.217 | | 0.167 | | 0.117 | |  | |  | |  | | 0.059 |  |
|  | | 129 | |  | |  | | 0.100 | | 0.333 | | 0.033 | |  | |  | |  | | 0.588 | 0.750 |
|  | | 131 | |  | |  | | 0.117 | | 0.083 | |  | |  | | 0.067 | |  | | 0.118 |  |
|  | | 133 | |  | |  | | 0.067 | |  | |  | |  | | 0.367 | | 0.417 | |  |  |
| T3-1T | | 226 | |  | |  | |  | |  | |  | | 0.029 | |  | |  | |  |  |
|  | | 230 | |  | |  | | 0.133 | |  | |  | | 0.129 | |  | |  | |  |  |
|  | | 234 | |  | |  | |  | |  | |  | | 0.243 | |  | |  | |  |  |
|  | | 238 | |  | |  | | 0.117 | | 0.083 | | 0.050 | | 0.114 | |  | |  | | 0.147 |  |
|  | | 242 | | 0.014 | |  | |  | | 0.083 | |  | | 0.343 | |  | |  | | 0.118 | 0.250 |
|  | | 246 | | 0.057 | |  | | 0.017 | |  | |  | | 0.086 | |  | |  | | 0.118 | 0.250 |
|  | | 250 | | 0.071 | |  | | 0.017 | |  | | 0.100 | | 0.057 | |  | |  | | 0.147 | 0.250 |
|  | | 254 | | 0.114 | |  | |  | |  | | 0.017 | |  | |  | |  | | 0.176 |  |
|  | | 256 | |  | |  | |  | |  | |  | |  | |  | |  | | 0.029 |  |
|  | | 258 | | 0.071 | |  | | 0.033 | |  | |  | |  | |  | |  | |  |  |
|  | | 262 | | 0.057 | | 0.033 | | 0.067 | |  | |  | |  | |  | |  | | 0.206 | 0.250 |
|  | | 266 | | 0.043 | |  | | 0.017 | | 0.083 | |  | |  | |  | |  | | 0.029 |  |
|  | | 270 | | 0.057 | |  | | 0.033 | |  | |  | |  | |  | |  | |  |  |
|  | | 274 | | 0.043 | |  | |  | |  | |  | |  | |  | |  | |  |  |
|  | | 278 | | 0.029 | |  | |  | |  | |  | |  | |  | |  | |  |  |
|  | | 282 | | 0.043 | |  | | 0.017 | |  | |  | |  | |  | |  | |  |  |
|  | | 286 | | 0.014 | |  | | 0.067 | |  | |  | |  | |  | |  | | 0.029 |  |
|  | | 290 | |  | |  | |  | | 0.500 | |  | |  | |  | |  | |  |  |
|  | | 294 | | 0.014 | | 0.033 | | 0.033 | |  | |  | |  | | 0.017 | | 0.139 | |  |  |
|  | | 298 | | 0.100 | | 0.017 | | 0.333 | | 0.083 | | 0.017 | |  | | 0.133 | |  | |  |  |
|  | | 302 | | 0.071 | | 0.217 | | 0.033 | |  | | 0.250 | |  | | 0.167 | | 0.181 | |  |  |
|  | | 306 | | 0.086 | | 0.017 | |  | | 0.083 | | 0.100 | |  | | 0.283 | | 0.278 | |  |  |
|  | | 310 | | 0.014 | | 0.350 | |  | |  | | 0.033 | |  | | 0.083 | | 0.319 | |  |  |
|  | | 312 | | 0.014 | |  | |  | |  | |  | |  | |  | |  | |  |  |
|  | | 314 | | 0.029 | | 0.233 | |  | | 0.083 | |  | |  | | 0.233 | | 0.042 | |  |  |
|  | | 318 | |  | | 0.017 | |  | |  | |  | |  | | 0.050 | | 0.042 | |  |  |
|  | | 322 | | 0.014 | | 0.017 | |  | |  | |  | |  | | 0.033 | |  | |  |  |
|  | | 326 | | 0.014 | |  | |  | |  | | 0.133 | |  | |  | |  | |  |  |
|  | | 332 | | 0.014 | | 0.067 | |  | |  | | 0.067 | |  | |  | |  | |  |  |
|  | | 336 | |  | |  | | 0.083 | |  | | 0.200 | |  | |  | |  | |  |  |
|  | | 340 | |  | |  | |  | |  | | 0.033 | |  | |  | |  | |  |  |
|  | | 344 | | 0.014 | |  | |  | |  | |  | |  | |  | |  | |  |  |
| T31-1 | | 100 | |  | |  | |  | |  | |  | |  | |  | |  | | 0.156 |  |
|  | | 106 | |  | |  | | 0.133 | | 0.250 | |  | | 0.057 | |  | |  | |  |  |
|  | | 108 | |  | |  | | 0.350 | | 0.333 | |  | | 0.657 | |  | |  | | 0.063 |  |
|  | | 110 | | 0.042 | |  | | 0.167 | | 0.083 | |  | |  | |  | |  | |  |  |
|  | | 112 | | 0.028 | | 0.172 | |  | |  | |  | | 0.214 | | 0.050 | | 0.417 | |  |  |
|  | | 114 | | 0.264 | | 0.224 | |  | | 0.167 | |  | | 0.071 | | 0.033 | |  | |  |  |
|  | | 116 | | 0.361 | | 0.603 | | 0.200 | | 0.083 | |  | |  | | 0.917 | | 0.583 | |  |  |
|  | | 118 | | 0.153 | |  | | 0.150 | |  | |  | |  | |  | |  | | 0.094 |  |
|  | | 120 | | 0.014 | |  | |  | | 0.083 | |  | |  | |  | |  | | 0.188 |  |
|  | | 122 | | 0.139 | |  | |  | |  | |  | |  | |  | |  | | 0.063 |  |
|  | | 126 | |  | |  | |  | |  | |  | |  | |  | |  | | 0.094 | 0.750 |
|  | | 128 | |  | |  | |  | |  | | 0.150 | |  | |  | |  | |  |  |
|  | | 130 | |  | |  | |  | |  | | 0.050 | |  | |  | |  | | 0.156 | 0.250 |
|  | | 132 | |  | |  | |  | |  | | 0.767 | |  | |  | |  | |  |  |
|  | | 134 | |  | |  | |  | |  | | 0.033 | |  | |  | |  | |  |  |
|  | | 136 | |  | |  | |  | |  | |  | |  | |  | |  | | 0.031 |  |
|  | | 138 | |  | |  | |  | |  | |  | |  | |  | |  | | 0.094 |  |
|  | | 140 | |  | |  | |  | |  | |  | |  | |  | |  | | 0.063 |  |
| T46-5 | | 146 | | 0.014 | | 0.133 | |  | |  | |  | |  | |  | |  | |  |  |
|  | | 150 | |  | |  | | 0.033 | | 0.083 | |  | |  | |  | |  | |  |  |
|  | | 154 | | 0.014 | |  | |  | | 0.083 | |  | |  | |  | |  | |  |  |
|  | | 158 | |  | |  | | 0.033 | | 0.250 | |  | |  | | 0.033 | |  | |  |  |
|  | | 162 | | 0.056 | |  | | 0.117 | |  | | 0.183 | |  | | 0.183 | | 0.056 | | 0.031 |  |
|  | | 164 | |  | |  | | 0.050 | |  | |  | |  | |  | |  | |  |  |
|  | | 166 | | 0.097 | | 0.217 | | 0.100 | |  | | 0.350 | | 0.132 | | 0.133 | | 0.542 | | 0.375 |  |
|  | | 168 | |  | |  | | 0.050 | |  | |  | |  | |  | |  | |  |  |
|  | | 170 | | 0.208 | | 0.267 | | 0.267 | | 0.250 | |  | | 0.191 | | 0.350 | | 0.403 | | 0.125 | 0.500 |
|  | | 172 | |  | |  | | 0.017 | |  | |  | |  | |  | |  | |  |  |
|  | | 174 | | 0.417 | | 0.333 | | 0.217 | | 0.250 | | 0.333 | | 0.397 | | 0.167 | |  | | 0.188 | 0.500 |
|  | | 178 | | 0.153 | | 0.050 | | 0.050 | | 0.083 | | 0.067 | | 0.250 | | 0.133 | |  | | 0.125 |  |
|  | | 182 | | 0.042 | |  | |  | |  | | 0.067 | | 0.029 | |  | |  | | 0.031 |  |
|  | | 186 | |  | |  | |  | |  | |  | |  | |  | |  | | 0.094 |  |
|  | | 190 | |  | |  | | 0.067 | |  | |  | |  | |  | |  | | 0.031 |  |
| Me1 | 168 | |  | |  | | 0.167 | | 0.083 | |  | | 0.429 | |  | |  | |  | |  |
|  | 170 | |  | |  | | 0.233 | | 0.083 | |  | | 0.243 | | 0.867 | | 0.722 | | 0.529 | |  |
|  | 172 | | 0.083 | | 0.083 | | 0.117 | |  | | 0.983 | | 0.029 | | 0.100 | |  | |  | |  |
|  | 174 | | 0.042 | | 0.433 | | 0.100 | | 0.083 | | 0.017 | |  | |  | |  | |  | |  |
|  | 176 | | 0.069 | | 0.100 | | 0.033 | | 0.167 | |  | |  | |  | |  | |  | |  |
|  | 178 | | 0.056 | | 0.050 | | 0.033 | | 0.083 | |  | | 0.214 | | 0.033 | |  | |  | |  |
|  | 180 | | 0.056 | | 0.117 | | 0.067 | | 0.333 | |  | | 0.086 | |  | | 0.278 | |  | | 1.000 |
|  | 182 | | 0.083 | |  | |  | | 0.083 | |  | |  | |  | |  | |  | |  |
|  | 184 | | 0.153 | |  | | 0.100 | | 0.083 | |  | |  | |  | |  | | 0.206 | |  |
|  | 186 | | 0.250 | |  | |  | |  | |  | |  | |  | |  | |  | |  |
|  | 188 | | 0.097 | | 0.083 | | 0.050 | |  | |  | |  | |  | |  | | 0.265 | |  |
|  | 190 | | 0.083 | | 0.133 | | 0.067 | |  | |  | |  | |  | |  | |  | |  |
|  | 192 | | 0.014 | |  | |  | |  | |  | |  | |  | |  | |  | |  |
|  | 194 | | 0.014 | |  | | 0.033 | |  | |  | |  | |  | |  | |  | |  |
| Me15 | 245 | | 0.076 | | 0.083 | |  | |  | |  | |  | |  | |  | |  | |  |
|  | 247 | |  | |  | | 0.033 | |  | |  | |  | |  | |  | |  | |  |
|  | 253 | |  | |  | | 0.017 | |  | |  | |  | |  | |  | |  | |  |
|  | 255 | |  | |  | | 0.117 | |  | |  | |  | |  | |  | |  | |  |
|  | 261 | |  | |  | |  | | 0.167 | |  | |  | |  | |  | |  | |  |
|  | 265 | |  | |  | | 0.167 | |  | |  | |  | | 0.017 | |  | |  | |  |
|  | 267 | |  | |  | | 0.033 | | 0.167 | |  | | 0.071 | | 0.948 | | 1.000 | |  | |  |
|  | 269 | | 0.076 | | 0.167 | | 0.067 | |  | |  | | 0.229 | | 0.017 | |  | |  | | 0.750 |
|  | 271 | | 0.121 | |  | | 0.150 | |  | | 0.400 | |  | | 0.017 | |  | |  | | 0.250 |
|  | 273 | | 0.030 | | 0.233 | | 0.117 | | 0.167 | | 0.133 | | 0.014 | |  | |  | | 0.567 | |  |
|  | 275 | | 0.015 | |  | | 0.033 | | 0.167 | | 0.467 | | 0.686 | |  | |  | | 0.233 | |  |
|  | 277 | |  | |  | | 0.067 | | 0.167 | |  | |  | |  | |  | |  | |  |
|  | 279 | | 0.045 | |  | | 0.200 | |  | |  | |  | |  | |  | |  | |  |
|  | 281 | | 0.030 | | 0.183 | |  | | 0.167 | |  | |  | |  | |  | | 0.200 | |  |
|  | 283 | | 0.045 | |  | |  | |  | |  | |  | |  | |  | |  | |  |
|  | 285 | | 0.152 | | 0.333 | |  | |  | |  | |  | |  | |  | |  | |  |
|  | 287 | | 0.364 | |  | |  | |  | |  | |  | |  | |  | |  | |  |
|  | 289 | | 0.015 | |  | |  | |  | |  | |  | |  | |  | |  | |  |
|  | 293 | | 0.030 | |  | |  | |  | |  | |  | |  | |  | |  | |  |
| Me16 | 260 | |  | |  | | 0.033 | |  | |  | |  | |  | |  | |  | |  |
|  | 262 | | 0.029 | |  | |  | |  | |  | |  | |  | |  | |  | |  |
|  | 264 | | 0.015 | |  | |  | |  | |  | |  | |  | |  | |  | |  |
|  | 266 | | 0.191 | | 0.467 | |  | |  | |  | |  | |  | |  | |  | |  |
|  | 268 | | 0.221 | | 0.183 | | 0.033 | |  | | 0.018 | |  | | 0.276 | | 0.667 | |  | |  |
|  | 270 | | 0.191 | | 0.033 | | 0.117 | | 0.250 | | 0.536 | |  | | 0.017 | |  | |  | |  |
|  | 272 | | 0.132 | | 0.050 | | 0.133 | |  | | 0.054 | | 0.468 | |  | |  | | 1.000 | |  |
|  | 274 | | 0.059 | | 0.133 | | 0.083 | | 0.125 | |  | | 0.016 | | 0.655 | | 0.333 | |  | |  |
|  | 276 | | 0.015 | |  | | 0.217 | | 0.500 | |  | | 0.129 | | 0.052 | |  | |  | |  |
|  | 278 | |  | |  | | 0.200 | |  | |  | | 0.097 | |  | |  | |  | | 1.000 |
|  | 280 | |  | |  | | 0.017 | |  | |  | | 0.258 | |  | |  | |  | |  |
|  | 282 | |  | |  | | 0.050 | |  | |  | | 0.032 | |  | |  | |  | |  |
|  | 284 | | 0.029 | | 0.133 | | 0.117 | |  | | 0.232 | |  | |  | |  | |  | |  |
|  | 286 | | 0.103 | |  | |  | | 0.125 | |  | |  | |  | |  | |  | |  |
|  | 288 | | 0.015 | |  | |  | |  | | 0.161 | |  | |  | |  | |  | |  |
| Me17 | 137 | | 0.083 | | 0.167 | |  | |  | |  | |  | |  | |  | |  | |  |
|  | 139 | | 0.250 | | 0.800 | | 0.050 | |  | |  | |  | |  | |  | |  | |  |
|  | 141 | | 0.556 | | 0.033 | |  | | 0.200 | |  | |  | |  | |  | |  | |  |
|  | 143 | | 0.028 | |  | | 0.050 | | 0.200 | | 0.200 | |  | |  | |  | |  | |  |
|  | 145 | | 0.014 | |  | | 0.083 | |  | | 0.017 | |  | |  | |  | |  | |  |
|  | 147 | | 0.014 | |  | | 0.217 | | 0.200 | | 0.367 | |  | | 0.150 | | 0.028 | | 1.000 | | 1.000 |
|  | 149 | | 0.056 | |  | | 0.367 | |  | |  | | 0.414 | |  | |  | |  | |  |
|  | 151 | |  | |  | | 0.150 | | 0.100 | |  | |  | | 0.117 | | 0.028 | |  | |  |
|  | 153 | |  | |  | | 0.033 | | 0.100 | | 0.333 | | 0.586 | | 0.733 | | 0.944 | |  | |  |
|  | 155 | |  | |  | | 0.033 | | 0.100 | | 0.083 | |  | |  | |  | |  | |  |
|  | 159 | |  | |  | | 0.017 | |  | |  | |  | |  | |  | |  | |  |
|  | 161 | |  | |  | |  | | 0.100 | |  | |  | |  | |  | |  | |  |
| Me28 | 162 | |  | |  | | 0.083 | |  | |  | |  | |  | |  | |  | |  |
|  | 168 | |  | |  | | 0.067 | | 0.500 | |  | |  | |  | |  | |  | |  |
|  | 170 | |  | |  | | 0.100 | | 0.167 | |  | |  | | 0.103 | | 0.153 | |  | |  |
|  | 172 | |  | |  | | 0.067 | |  | |  | |  | |  | |  | |  | |  |
|  | 174 | |  | |  | | 0.067 | |  | |  | |  | |  | |  | |  | |  |
|  | 176 | |  | |  | |  | |  | |  | | 0.086 | |  | |  | |  | |  |
|  | 178 | | 0.111 | | 0.017 | |  | |  | |  | | 0.029 | | 0.655 | | 0.250 | |  | |  |
|  | 180 | | 0.069 | | 0.450 | |  | |  | |  | |  | | 0.224 | | 0.403 | |  | |  |
|  | 182 | | 0.083 | |  | | 0.017 | |  | |  | |  | | 0.017 | | 0.194 | |  | |  |
|  | 184 | | 0.069 | |  | | 0.083 | |  | |  | |  | |  | |  | | 0.067 | |  |
|  | 186 | | 0.083 | |  | | 0.017 | |  | |  | |  | |  | |  | | 0.333 | | 1.000 |
|  | 188 | | 0.014 | | 0.117 | |  | |  | |  | |  | |  | |  | |  | |  |
|  | 190 | | 0.014 | |  | | 0.133 | | 0.083 | |  | |  | |  | |  | | 0.100 | |  |
|  | 192 | | 0.097 | |  | | 0.033 | |  | | 0.267 | |  | |  | |  | |  | |  |
|  | 194 | | 0.042 | | 0.300 | | 0.017 | |  | | 0.017 | |  | |  | |  | | 0.067 | |  |
|  | 196 | | 0.111 | |  | | 0.083 | |  | | 0.183 | | 0.157 | |  | |  | |  | |  |
|  | 198 | | 0.125 | | 0.117 | | 0.050 | |  | | 0.283 | | 0.057 | |  | |  | |  | |  |
|  | 200 | | 0.042 | |  | | 0.017 | |  | | 0.200 | | 0.271 | |  | |  | |  | |  |
|  | 202 | | 0.042 | |  | |  | |  | | 0.017 | | 0.114 | |  | |  | |  | |  |
|  | 204 | | 0.028 | |  | | 0.117 | |  | | 0.033 | | 0.143 | |  | |  | |  | |  |
|  | 206 | | 0.028 | |  | |  | | 0.250 | |  | | 0.043 | |  | |  | | 0.167 | |  |
|  | 208 | | 0.014 | |  | |  | |  | |  | | 0.071 | |  | |  | | 0.200 | |  |
|  | 210 | | 0.014 | |  | |  | |  | |  | |  | |  | |  | | 0.067 | |  |
|  | 212 | | 0.014 | |  | |  | |  | |  | | 0.029 | |  | |  | |  | |  |
|  | 222 | |  | |  | | 0.050 | |  | |  | |  | |  | |  | |  | |  |
| T15.1 | 169 | |  | |  | |  | |  | |  | |  | | 0.767 | | 0.472 | |  | |  |
|  | 171 | |  | |  | | 0.100 | |  | |  | |  | | 0.233 | | 0.528 | |  | |  |
|  | 173 | | 0.029 | |  | | 0.100 | | 0.500 | |  | |  | |  | |  | |  | |  |
|  | 175 | | 0.235 | | 0.717 | | 0.233 | | 0.400 | |  | | 0.871 | |  | |  | |  | | 0.750 |
|  | 177 | | 0.250 | |  | | 0.500 | |  | |  | | 0.129 | |  | |  | |  | | 0.250 |
|  | 179 | | 0.029 | |  | | 0.067 | |  | | 1.000 | |  | |  | |  | |  | |  |
|  | 183 | | 0.309 | | 0.267 | |  | |  | |  | |  | |  | |  | | 0.882 | |  |
|  | 185 | | 0.147 | | 0.017 | |  | | 0.100 | |  | |  | |  | |  | | 0.118 | |  |
| T32.1 | 149 | |  | |  | |  | |  | |  | |  | | 0.138 | |  | |  | |  |
|  | 161 | | 0.736 | | 0.867 | |  | |  | |  | |  | |  | |  | |  | |  |
|  | 163 | | 0.264 | | 0.133 | |  | |  | |  | |  | |  | |  | |  | |  |
|  | 175 | |  | |  | | 0.217 | |  | | 0.033 | |  | | 0.414 | | 0.042 | |  | |  |
|  | 177 | |  | |  | | 0.400 | | 0.375 | | 0.933 | | 0.014 | | 0.448 | | 0.958 | | 1.000 | | 1.000 |
|  | 181 | |  | |  | | 0.067 | | 0.500 | |  | | 0.986 | |  | |  | |  | |  |
|  | 183 | |  | |  | | 0.150 | |  | |  | |  | |  | |  | |  | |  |
|  | 185 | |  | |  | | 0.067 | |  | |  | |  | |  | |  | |  | |  |
|  | 187 | |  | |  | | 0.100 | | 0.125 | | 0.033 | |  | |  | |  | |  | |  |

KI = Kangaroo Island; KwI = Kawau Island, New Zealand; Tut = Tutanning; Per = Perup; GI = Garden Island; EWI = East Wallabi Island; WWI = West Wallabi Island; NI = North Island; MI = Middle Island; NTP = North Twin Peak Island.
