# Supplementary material for: Extensive genetic differentiation detected within a model marsupial, the tammar wallaby (Notamacropus eugenii)
Source: PLoS One. 2017 Mar 3;12(3):e0172777. doi: 10.1371/journal.pone.0172777 (PMC5336229; doi:10.1371/journal.pone.0172777)
Supplement: S3 Table — KI = Kangaroo Island; KwI = Kawau Island, New Zealand; Tut = Tutanning; Per = Perup; GI = Garden Island; EWI = East Wallabi Island; WWI = West Wallabi Island; NI = North Island; MI = Middle Island. (DOCX) [file pone.0172777.s003.docx]

### S2 Table. Allelic combinations of the 32 Y haplotypes identified in nine tammar wallaby (*Notamacropus eugenii*) populations.

| **Y Haplotype** | **Population** | **Locus** | | | |
| --- | --- | --- | --- | --- | --- |
|  |  | **Y01** | **Y28** | **Y37A** | **Y37B** |
| 1 | KI, KwI | 322 | 335 | 155 | 159 |
| 2 | KI, KwI | 322 | 335 | 155 | 161 |
| 3 | KI, KwI | 318 | 343 | 155 | 159 |
| 4 | KI | 318 | 335 | 155 | 159 |
| 5 | KI | 328 | 341 | 155 | 159 |
| 6 | KI | 318 | 347 | 155 | 159 |
| 7 | KI | 318 | 335 | 155 | 161 |
| 8 | KI | 320 | 335 | 155 | 159 |
| 9 | KI | 320 | 341 | 155 | 159 |
| 10 | KI | 324 | 341 | 155 | 159 |
| 11 | KI | 324 | 337 | 155 | 159 |
| 12 | KI | 324 | 343 | 155 | 159 |
| 13 | KI | 326 | 335 | 155 | 159 |
| 14 | KI | 312 | 347 | 155 | 159 |
| 15 | KI | 330 | 347 | 155 | 159 |
| 16 | KI | 318 | 341 | 155 | 159 |
| 17 | KwI | 320 | 335 | 155 | 161 |
| 18 | KwI | 322 | 343 | 155 | 159 |
| 19 | Tut | 308 | 331 | 151 | 169 |
| 20 | Tut | 308 | 335 | 151 | 169 |
| 21 | Tut | 308 | 337 | 151 | 163 |
| 22 | Tut | 308 | 329 | 151 | 169 |
| 23 | Tut | 308 | 331 | 151 | 163 |
| 24 | Tut | 308 | 337 | 151 | 169 |
| 25 | Per | 308 | 335 | 155 | 169 |
| 26 | GI | 308 | 325 | 151 | 167 |
| 27 | EWI | 310 | 325 | 151 | 169 |
| 28 | WWI, NI | 314 | 331 | 151 | 169 |
| 29 | WWI | 314 | 329 | 151 | 169 |
| 30 | MI | 306 | 335 | 151 | 167 |
| 31 | MI | 306 | 325 | 151 | 167 |
| 32 | MI | 310 | 335 | 151 | 167 |

KI = Kangaroo Island; KwI = Kawau Island, New Zealand; Tut = Tutanning; Per = Perup; GI = Garden Island; EWI = East Wallabi Island; WWI = West Wallabi Island; NI = North Island; MI = Middle Island.
