# Supplementary figures and images for: Extensive genetic differentiation detected within a model marsupial, the tammar wallaby (Notamacropus eugenii)
Source: PLoS One. 2017 Mar 3;12(3):e0172777. doi: 10.1371/journal.pone.0172777 (PMC5336229; doi:10.1371/journal.pone.0172777)

**S1 Fig**. **Structure output showing a) maximum *L(K)* at K = 7 and b) maximum Δ*K* at K = 8 (b).**

**a)**


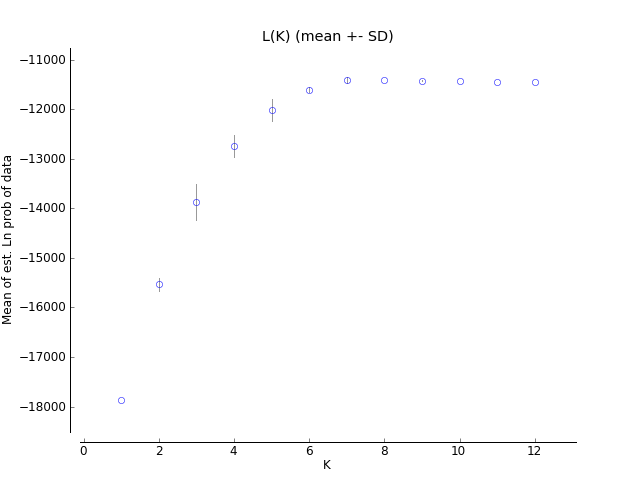


**b)**


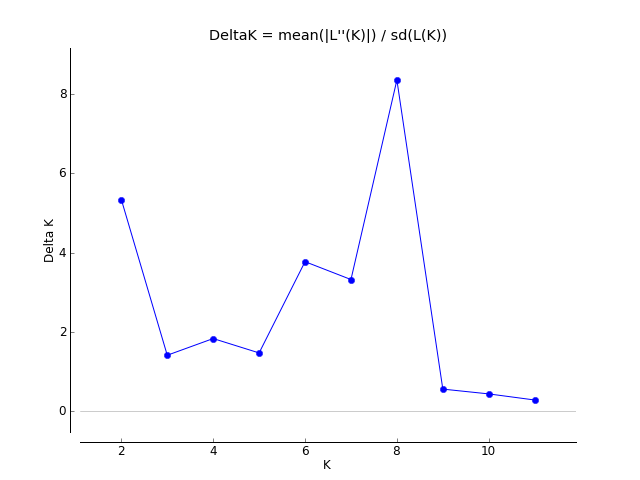

Supplement: S1 Fig — (DOCX) [file pone.0172777.s004.docx]
